# A Dual-Polarimetric SAR Ship Detection Dataset and a Memory-Augmented Autoencoder-Based Detection Method

**DOI:** 10.3390/s21248478

**Published:** 2021-12-19

**Authors:** Yuxin Hu, Yini Li, Zongxu Pan

**Affiliations:** 1 Aerospace Information Research Institute, Chinese Academy of Sciences, Beijing 100094, China; huyx@aircas.ac.cn (Y.H.); liyini19@mails.ucas.ac.cn (Y.L.); 2 Key Laboratory of Technology in Geo-Spatial Information Processing and Application System, Chinese Academy of Sciences, Beijing 100094, China; 3 School of Electronic, Electrical and Communication Engineering, University of Chinese Academy of Sciences, Beijing 101408, China

**Keywords:** ship detection, dual-polarimetric dataset, pseudo-color enhancement, autoencoder based anomaly detection

## Abstract

With the development of imaging and space-borne satellite technology, a growing number of multipolarized SAR imageries have been implemented for object detection. However, most of the existing public SAR ship datasets are grayscale images under single polarization mode. To make full use of the polarization characteristics of multipolarized SAR, a dual-polarimetric SAR dataset specifically used for ship detection is presented in this paper (DSSDD). For construction, 50 dual-polarimetric Sentinel-1 SAR images were cropped into 1236 image slices with the size of 256 × 256 pixels. The variances and covariance of both VV and VH polarization were fused into R,G,B channels of the pseudo-color image. Each ship was labeled with both a rotatable bounding box (RBox) and a horizontal bounding box (BBox). Apart from 8-bit pseudo-color images, DSSDD also provides 16-bit complex data for readers. Two prevalent object detectors R^3^Det and Yolo-v4 were implemented on DSSDD to establish the baselines of the detectors with the RBox and BBox respectively. Furthermore, we proposed a weakly supervised ship detection method based on anomaly detection via advanced memory-augmented autoencoder (MemAE), which can significantly remove false alarms generated by the two-parameter CFAR algorithm applied upon our dual-polarimetric dataset. The proposed advanced MemAE method has the advantages of a lower annotation workload, high efficiency, good performance even compared with supervised methods, making it a promising direction for ship detection in dual-polarimetric SAR images. The dataset is available on github.

## 1. Introduction

Accurate and realtime surveillance of marine vessels has great significance for both border safety and navigation management in the case of increasingly complex port traffic. [1,2] As an active microwave sensor, Synthetic Aperture Radar (SAR) [3] has the trait of observation targets under all-day and all-weather situations, which makes SAR play an important role in marine ship detection [1,2,4,5,6,7]. With the advances of imaging technology a large number of high-quality polarimetric SAR (PolSAR) images can be provided by global earth observation satellites [8], e.g., Sentinel-1 [9], as well as applied into automatic detection fields [10]. It has been verified that multipolarization imagery containing more object polarization features are more conducive to detection than single polarization ones [11,12,13]. Therefore, studying the issue of ship detection adopting PolSAR imagery has become a prevalent trend [7,10].

Throughout all the conventional approaches, ship detection is mainly based on manually selected characteristics, including polarization features, as well as statistical characteristics of background clutter [7,14,15,16]. The constant false alarm rate (CFAR) is commonly used for ship target detection in PolSAR images [4,17,18]. Typical CFAR methods take appropriate thresholds to filter target pixels [19,20]. Another kind of method is built on the theory of polarization decomposition [12,21], which derives polarization features from the scattering matrix or covariance matrix, [22,23,24] and has been applied to PolSAR ship detection effectively. Though conventional technologies have achieved good performance in ship detection, there exist some obvious limitations. One is that the amount of manually selected features is limited, resulting in the insufficient representation of ships. The other is that detection environments are limited, which may signify those methods lack generalization and robustness for ships near land or in complex sea backgrounds [2,6]. To meet the need for accuracy and efficiency, a new kind of algorithm based on deep learning provides clues for the field of detection.

Beneficial for its unique end-to-end hierarchical structure, the convolutional neural network (CNN) [25] demonstrates the powerful capability of automatic feature extraction of images [26]. R-CNN [27] first creatively applied CNN to detection. Fast R-CNN [28] and Faster R-CNN [29] have become classic two-stage target detection algorithms. In addition to two-stage methods, one-stage algorithms, e.g., SSD [30] and YOLO series [31,32,33,34], have better computing speed at the inference stage through abandoning the region proposed network (RPN) [29]. Since the Focal Loss [35] technique solved the imbalance between the amount of positive and negative samples, the precision and recall of the single-stage algorithm are comparable to that of the two-stage algorithm. Other proposed network frameworks like FPN [36], PANet [37], CSPNet [38] try to aggregate information from different hierarchies or stages. Much work has been done by researchers in the area of SAR imagery object detection [2,6]. Inspired by CMS-RCNN [39], Kang et al. proposed a context-based multilayer fusion network [40] to detect small ships. Liu et al. combined multiscale features with a rotating detection framework and proposed a single-stage detection network named DRBox [41]. Wei et al. exploited a high-resolution feature pyramid network (HRFPN) that connected multilayer subnetworks in parallel in HR-SDNet [42] to refine consequent outputs. All these studies have proved the effectiveness and robustness of deep CNN in SAR imagery detection.

The basic premise of deep learning is adequate training sets. As for the existing SAR ship datasets that most previous studies mainly relied on, most of them consist of single polarization images quantified to grayscale pixels. The commonly used SAR ship detection dataset (SSDD) [43] contains an insufficient number of objects, and the intensity of pixels ranges from 0 to 255 without the original data provided. OpenSARship [44] used for ship classification, constructed by SJTU, has 10 unbalanced categories, and original high precision data are included. Yet ship chips in OpenSARship have extremely small sizes, and the lack of scattering information could lead to bad performance on generalization. Compared to SSDD, a High-Resolution SAR Images Dataset (HRSID) [45] has an adequate number of chips that have been processed to 8-bit JPEG format with single polarization mode for one image, and most in co-polarization mode.

Pseudo-color enhancement is a common way to realize the visualization of PolSAR images. It can display small grayscale differences as distinct color differences, and effectively embody the polarization information of the target [46,47,48]. PolSAR pseudo-color enhancement is mostly used for image segmentation and classification [49,50,51,52]. As for the ship detection field, only a few works based on the deep neural network have been performed on pseudo-color enhanced data. Fan et al. trained the CNN framework by using a Pauli pseudo-color dataset composed of quad-polarization SAR [53]. Zou et al. synthesized pseudo-color images by taking three single-polarimetric SAR images obtained at continuous azimuth angles [54]. Unfortunately, these authors did not open their dataset to the public. As far as we know, there is still not an appropriate and accessible dual-polarimetric dataset for SAR ship detection in deep learning. Both covariance matrix C and coherence matrix T of multipolarization imagery contain all the polarization information [7]. Therefore, we utilize the specific value of the C or T matrix as different channels to generate pseudo-color images.

Among SAR satellites, Sentinel-1 in the European Space Agency’s Copernicus programme consists of two satellites carrying C-band radar, could provide continuous images, and has an accessible database [9]. To meet the need of quantity and quality, we selected dual vertical (DV) polarization images in Sentinel-1 IW mode as samples to construct pseudo-color enhancement and finally built this dual-polarimetric SAR ship detection dataset (DSSDD). Each pixel was obtained by taking $\left|C_{11}\right|,\;\left|C_{12}\right|,\;\left|C_{22}\right|$ elements of polarimetric covariance matrix as red, green, and blue channels respectively. When labeling ships, we employed both a rotatable bounding box (RBox) and horizontal bounding box (BBox) to represent objects more precisely. For the convenience of experiment execution, images were quantified as input for deep neural networks, but the original 16-bit data are reserved as well. All annotations and images in DSSDD are accessible online [55] at https://github.com/liyiniiecas/A_Dual-polarimetric_SAR_Ship_Detection_Dataset, accessed on 3 November 2021.

In addition, inspired by an anomaly detection algorithm named memory-augmented deep autoencoder (MemAE) [56], we proposed an advanced weakly supervised ship detection method that takes into account both computational cost and prediction accuracy. The former advantage comes from adopting the two-parameter CFAR method [18] for preliminary detection and the latter from MemAE for the further screening of targets. The CFAR detection is simple and effective and tends to have false alarms. We applied anomaly detection to eliminate abnormal targets after CFAR generated a region proposal and kept the real ship targets. Compared with CNN-based detectors, our method does not rely on massive networks nor high-performance equipment, which can mitigate computation burden and decrease the memory cost. We show the validity of this method on our dual-polarimetric dataset, as detailed below.

Our contributions are as follow:

1. A new open dual-polarimetric dataset based on dual-vertical polarization images was constructed. Labels of RBoxes and BBoxes are provided, respectively. Computation details and statistical analysis are described also. This could be the first dual-polarimetric dataset applied for deep neural network ship detection, which will hopefully boost the development of this area.

2. Two prevalent detection networks were adopted to build baselines of our dataset. Experimental results on SOTAs show that the pseudo-color processing method fused with multipolarization information had a better detection performance than the single polarization processing method.

3. A ship detection algorithm based on anomaly detection is proposed achieving superior detected results than conventional methods. Its validity is demonstrated on our DSSDD.

This paper is organized as follows. Section 2 describes the build process and properties of DSSDD. Then we present baselines of two typical networks in Section 3. Our weakly supervised detection method is proposed in Section 4. Section 5 concludes this paper.

## 2. The Construction of the Dataset

### 2.1. The Original SAR Imageries

To ensure the quantity and quality of our ship-specific interpretation dataset, 50 Level-1 Sentinel-1 Interferometric Wide swath (IW) mode imageries were selected as original construction data. According to the Sentinel-1 official guide provided by European Space Agency (ESA), IW mode captures three sub-swaths using Terrain Observation with Progressive Scans SAR (TOPSAR), and each sub-swath contains a total of nine bursts, where each burst has been processed as a separate single look complex (SLC) image [9].

VV co-polarization and VH cross-polarization products are generated under IW mode. The cross-polarization scattering has stronger energy intensity than the co-polarization scattering, thus, the shape and skeleton of ships are clearer. On the other side, the inshore scattering and sea clutter noise of cross-polarization are stronger than that of co-polarization [45]. Under the radar pulse emitted by the sensor, the ships appear as spindle-shaped bright pixels at a double reflection. Other details including swath and incident angle are depicted in Table 1. It is worth noting that the resolution in range and azimuth were different from that of the data after preprocessing.

**Figure 1:**
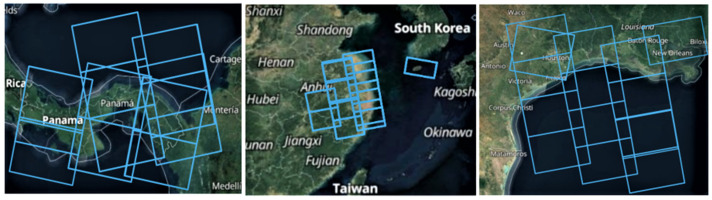
Representative regions acquired from the Sentinel-1 IW. Blue boxes indicate the coverage of each image.

We selected the regions at large ports and busy sea areas with numerous ships as well as specific scenes to acquire typical and sufficient samples. Figure 1 presents some wide swath coverage of our datasets, including Shanghai, the Suez Canal, the Gulf of Mexico, Port of Houston, the Strait of Gibraltar, etc. All the original images with wide swath were downloaded from the official website [57].

### 2.2. Preprocessing for SAR Imageries

**Figure 2:**
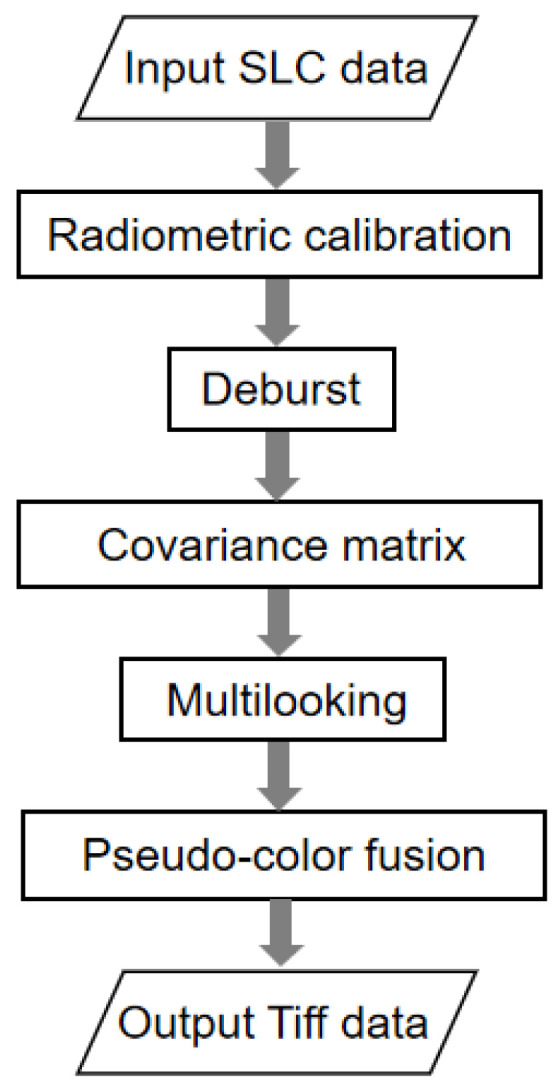
Preprocessing flow of the original Sentinel-1 imagery.

Level-1 SLC data comprising complex imagery with amplitude and phase were preprocessed before we constructed the dataset. SNAP 8.0 [58] was employed in our experiment to operate the radiometric calibration, multilooking, deburst, and matrix calculations. The complete procedure is shown in Figure 2.

Radiometric calibration was the crucial measure to enhance the geometric radiation quality of spaceborne SAR by eliminating imaging errors through correcting spectral reflectance or the spectral radiation brightness of ground objects, which was the first step to be carried out.

TOPSAR products consist of a series of bursts as well as the strip between bursts without signal [9]. Deburst was employed for merging three sub-swaths and nine bursts within the sub-swath to create a complete product. TOPSAR technology significantly reduced scalloping effects compared to conventional scanning SAR.

PolSAR obtains the polarization characteristics of targets by measuring the Sinclair scattering matrix S [21]. The Sinclair scattering matrix is the key observation to display the relationship between the incident electromagnetic field vector and the scattering electromagnetic field vector [59] and is defined as (1). Under the dual-polarization mode with VV and VH, two non-zero elements $S_{vv}$ and $S_{vh}$ in S describe the echo voltage received. The remaining two elements $S_{hh}$ and $S_{hv}$ are equal to 0 under this circumstance.

(1) $$S=\left[\begin{array}{cc} S_{hh} & S_{hv}\\ S_{vh} & S_{vv} \end{array}\right]$$

The Lexicographic scattering vector $\vec{k_{L}}$ is obtained by straight order expansion on a completely orthogonal basis, which can be formulated as (2):

(2) $$\vec{k_{L}}={\left[\begin{array}{cc} S_{vh} & S_{vv} \end{array}\right]}^{T}$$

The polarimetric covariance matrix is derived from the Sinclair scattering matrix by calculating the Kronecker inner product of $\vec{k_{L}}$. The polarization covariance matrix $C_{2}$ is formulated in (3), where H refers to the conjugate transpose operation:

(3) $$C_{2}=\langle \vec{k_{L}}\cdot {\vec{k_{L}}}^{H}\rangle =\left[\begin{array}{cc} \langle {\left|S_{vh}\right|}^{2}\rangle & \langle S_{vh}S_{vv}{}^{*}\rangle \\ \langle S_{vv}S_{vh}{}^{*}\rangle & \langle {\left|S_{vv}\right|}^{2}\rangle \end{array}\right]=\left[\begin{array}{cc} C_{11} & C_{12}\\ C_{21} & C_{22} \end{array}\right]$$

From the expression of $C_{2}$ in (3), it is obvious that complex numbers $C_{12}$ and $C_{21}$ are conjugate, while $C_{11}$, $C_{22}$ representing the energy of VH polarization and VV polarization respectively are both real numbers. Accordingly, three elements $C_{11}$, $C_{12}$, $C_{22}$ were used to generate a pseudo-color image as they contained the entire polarization information. In brief, the absolute value of the three elements $C_{11}$, $C_{12}$, $C_{22}$ at each pixel were taken as three channels R, B, and G of a pseudo-color image.

**Figure 3:**
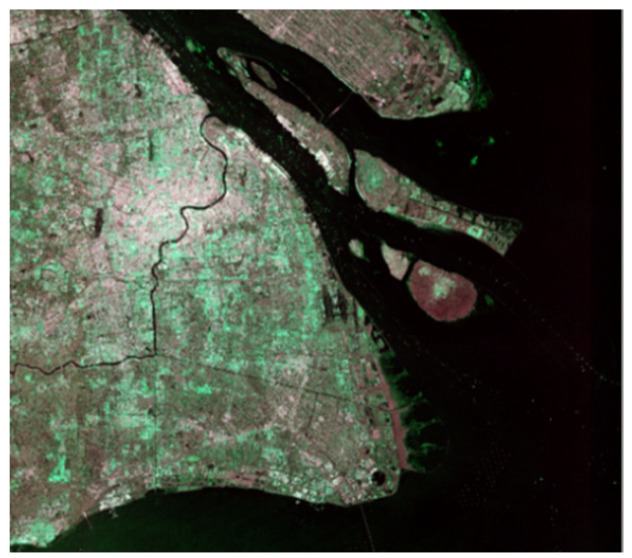
Example of 8-bit pseudo-color enhanced product after preprocessing.

For the range direction, the resolution was much smaller than that of azimuth, the image was stretched along the azimuth side. We adopted multilooking technology to obtain approximately square pixels in addition to eliminating and reducing speckle noise [60]. It is worth noting that, after multilooking processing, the range and azimuth resolution were converted to approximately 9 m × 14 m. Finally, as the output pixel was a 16-bit value that could not be displayed on screen directly, we compressed the data for convenience. SNAP 8.0 was adopted to automatically save the image into 8-bit format data and then complete the quantification, as the final products shown in Figure 3. All the following experiments were conduct using 8-bit format data.

### 2.3. Data Format

**Figure 4:**
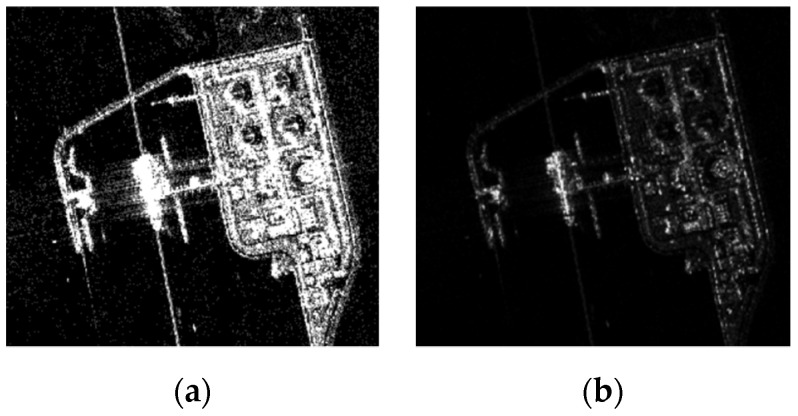
Illustration of improperly compressed SAR images; (**a**) is overexposed, and (**b**) is underexposed.

During the experiments, we found that the CNN input was 8-bit compressed SAR data. While the original polarization SAR data was characterized by being distributed in a high dynamic range, the compressing to the 0–255 range can cause information loss of SAR imagery [61]. As is the case with Figure 4, improper compression may lead to oveexposure or underexposure. Although researchers have proposed some dynamic range compression algorithms that alleviate the problems caused by compression to a certain extent, there are still some defects such as lack of details and poor adaptive processing capabilities. For data-driven detectors, the quality of images will directly affect the results of the detection. Therefore, in DSSDD, we provide additional data without quantification.

**Figure 5:**
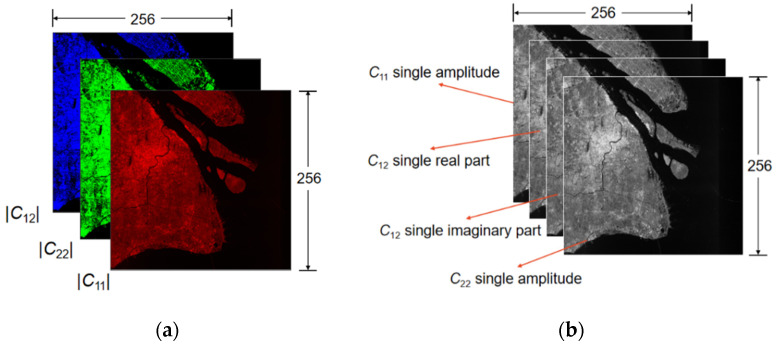
Two kinds of data formats in DSSDD: (**a**) Description of the 8-bit compressed pseudo-color images; and (**b**) Description of the 16-bit uncompressed images.

Two kinds of data formats are described in Figure 5. The 8-bit compressed slices input to the detector were stored in PNG format. In Figure 5a, the R, G, and B channels indicate the amplitude value of the covariance matrix elements $C_{11}$, $C_{12}$, and $C_{22}$. For convenience, the quantification was completed by an algorithm embedded in the SNAP 8.0.

Figure 5b is the 16-bit original data format containing phase information. The second and third channels indicate the imaginary part value and real part value of $C_{12}$, respectively. While the $C_{11}$ and $C_{22}$ are real numbers, they occupy the first and last channels, respectively.

### 2.4. Strategy for Labeling the Dataset

Due to the wide width of the IW image, the size of images processed in the previous section was larger than 10,000 × 10,000 pixels, which was not conducive to target annotation and data storage. Candidate sub-images were screened out after we cropped the original data with a 50-pixel overlap. Then, we used the label tool RoLabelImg [62] to mark rotating boxes and horizontal boxes on candidate sub-images. Last, the sub-images were cropped again with sliding windows to 256 × 256 ship slices and saved in PNG format. Hoping readers can take full advantage of our data, we also provide additional single-precision slices without quantification.

**Figure 6:**
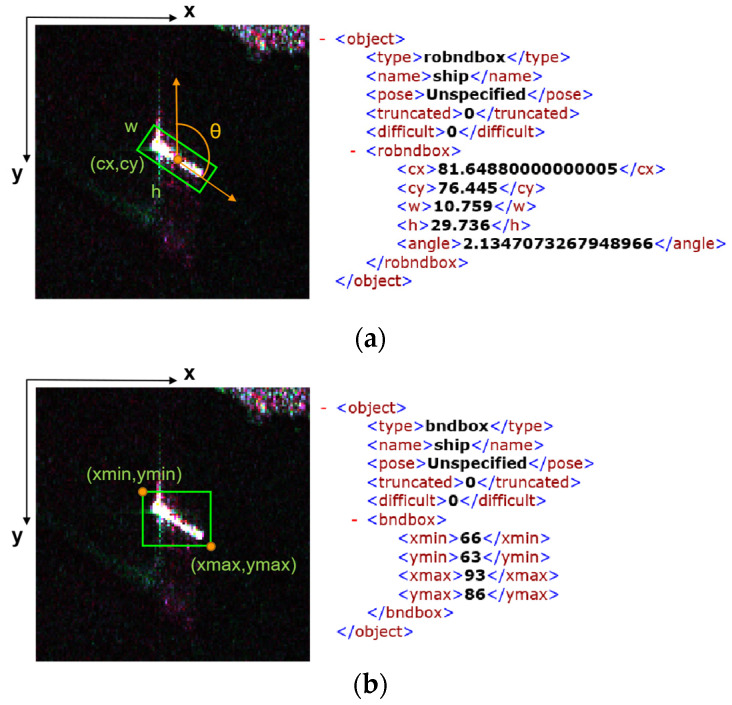
Ship slices are annotated as both RBoxes and BBoxes. (**a**) is detailed information of a RBox that represents a rotating rectangle; and (**b**) is detailed information of a BBox that represents a horizontal rectangle.

Each slice has a corresponding XML format annotation file, indicating the slice size, slice name, and annotation type. The RBox label is tagged as “robndbox”, where “cx”, “cy”, “w”, “h”, and “angle” indicate the center coordinates, height, width, and angle of a box, respectively. The range of rotatable angle is 0~Π, consistent with the height direction. Correspondingly, the BBox label is tagged as “bndbox”, where “xmin”, “xmax”, “ymin”, “ymax” refer to the top left corner and the lower right corner coordinates of a box, respectively; more details are shown in Figure 6 below.

### 2.5. Properties Analysis

Distinguished from optical images, SAR images receive part of the backward scattering energy from targets; the quality of SAR imaging varies with sea clutter, incident angle, and other external factors to some extent [45]. Except for targets in the calm and pelagic background, our dataset also involves such challenging and complicated scenarios as inshore and chaos clutter situations to achieve feature balance and add complexity.

**Figure 7:**
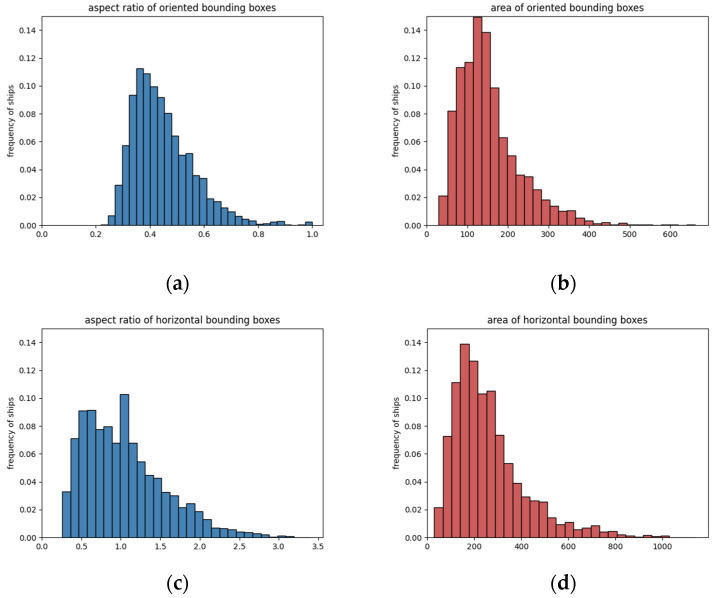
Statistical histograms of RBoxes and BBoxes in DSSDD. (**a**,**b**) illustrate the aspect ratio and area distribution of RBoxes; (**c**,**d**) illustrate the aspect ratio and area distribution of BBoxes.

After construction was completed, a total of 1236 image slices with 3540 ship targets were concluded. All images were randomly split into the training set (70% of all) and the test set (30% of all). It contained one category only for ship interpretation, whereas other categories appearing in the SAR images did not have annotations. We analyzed statistics by counting aspect ratio and area; both rotatable and rectangle boxes were taken into consideration and illustrated as a histogram in Figure 7. It can be seen from the histogram that the aspect ratio and area were approximately a Gaussian distribution within a certain range, which was also in line with our data expectations.

The aspect ratio refers to the ratio of box width and height, which has an impact on anchor box selection in CNN. For each RBox, the longer side is considered as height and the shorter side as width, thus its aspect ratio score was no more than 1. The aspect ratio of the BBox has no such limitation. The average aspect ratio of RBoxes was around 0.5 with more than 80% of that case being less than 0.6. Up to 90% of the BBoxes were concentrated from 0.5 to 2 in terms of the aspect ratio. As for the area, which refers to the number of pixels a box occupied, the area of an RBox was smaller than or equal to that of the same object’s BBox. The average area of the RBoxes was 154, less than that of the BBoxes at 251. The maximum area of the RBoxes would not exceed 700 pixels and 1200 pixels for BBoxes. In MS COCO [63] evaluation metrics, objects were divided into small, medium, and large scale by area, where an object having less than a 32 × 32 pixels area corresponded to the small object. According to this standard, our dataset had comparatively fewer large targets, while small objects were up to 98% in the entire dataset, as high-resolution Sentinel-1 images are infrequent. Small object detection was an emphasis during construction. The characteristic of small objects leads to the tendency of fewer features and targets omission when detecting, and it is also the challenge faced by our research.

**Figure 8:**
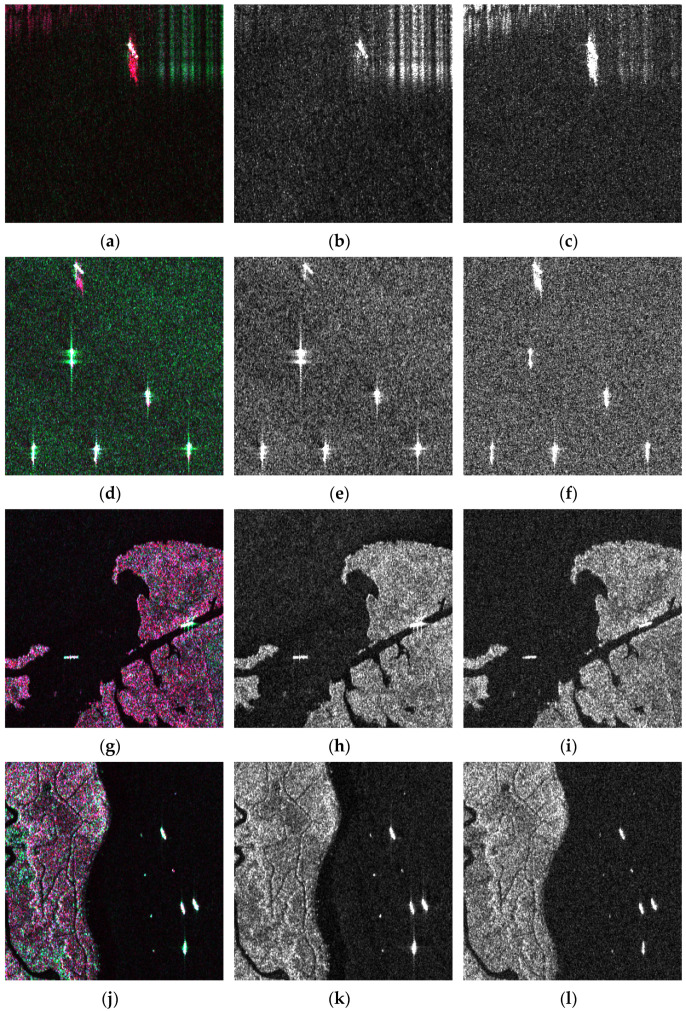
Comparison of VV and VH polarized images with corresponding DSSDD data. (**a**,**d**,**g**,**j**) are pseudo-color maps, (**b**,**e**,**h**,**k**) are VV polarized images, and (**c**,**f**,**i**,**l**) are VH polarized data. Our pseudo-color image had the advantage of weakening the sidelobe and land noise on hulls and clarified the ship skeleton.

Compared with existing SAR ship datasets quantified to grayscale, our pseudo-color images had the advantage of weakening the sidelobe and land noise on hulls and clarifying the ship skeleton. We present the comparison of VV and VH images with our color slices in Figure 8, where Figure 8a,d,g,j are enhanced data, Figure 8b,e,h,k are VV polarized images, and Figure 8c,f,i,l are VH polarized. For the impact of the Doppler shift [64], the coherent superposition of backscattering produced considerable cross-sidelobe, which seriously contaminated the morphology and features of objects. High sidelobe would notably diminish the ability to recognize corner cases and interpret easy cases accurately as well. Sidelobe and land noise perform divergent angles and intensity under different polarization modes. In addition, ships have nearly identical white pixels due to double reflections from metal decks [65]. As shown in Figure 8a,d, the sidelobe is a pink or green radial pattern, and deck scattering appears as a brightly white fusiform strip. However, the ship and sidelobe appear almost the same brightness in Figure 8b,c,e,f. The pseudo-color enhancement did not actually change pixels’ grayscale but rather changed the color, which also retained the complete skeleton of the ship. In Figure 8h,i,k,l, the pixels of land and ship had similar brightness, especially in Figure 8i the object on the right side almost blended into the land. But in Figure 8g,j, the reflection brightness of the coarse land surface was suppressed, and the silhouette of the hulls was more prominent. Merging different polarization patterns can relieve detrimental interference without spoiling ship bodies.

## 3. Detection Benchmarks of Supervised Approaches

### 3.1. Benchmark Networks

CNN-based detectors are generally divided into two categories: single-stage detectors and two-stage detectors, which have the backbone network of feature extraction, bounding box regression, and category classification branches [26]. A two-stage network has additional RPN to propose candidate boxes for the following prediction, which greatly increases the time cost of detection.

Recently, some tricks such as hard sample selection or Focal Loss were proposed to prevent models from being dominated by easy cases, since the imbalance between positive and negative samples limits the network’s ability to learn corner cases. A single-stage detection algorithm has become mainstream. Among these, YOLOv4 [34] reached 43.5% AP on MS COCO, which was superior to other SOTA detectors in comprehensive consideration of accuracy and speed. For the above reason, we chose YOLOv4 as the baseline for detectors with BBox.

Remote sensing images have scenes including plenty of small and arbitrarily-oriented objects. In such a situation, BBox has a defect that each box envelopes parts of other targets, which results in boxes with a high overlap degree being prone to be suppressed during Non-Maximum Suppression (NMS) [66]. RBoxes was introduced to overcome the drawbacks of BBoxes.

**Figure 9:**
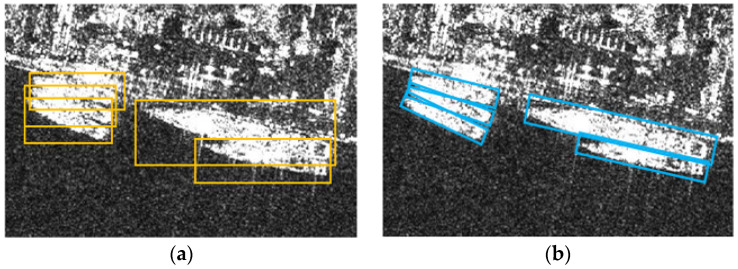
Comparison of RBox and BBox. (**a**) illustrates BBoxes representing targets; (**b**) illustrates RBoxes representing targets.

As shown in Figure 9, RBox has superiority in describing densely arranged objects and excluding background interference for having one more hyperparameter. Consequently, the RBox detector is still the most robust choice in the field of remote sensing object detection. R^3^det [67] is an accurate and fast-rotating target detector. Extensive experiments on DOTA [68], HRSC2016 [69], and ICDAR2015 [70] datasets have shown the excellent performance of this detector. Therefore, R^3^Det served as the baseline for RBox detectors.

#### 3.1.1. R^3^Det

**Figure 10:**
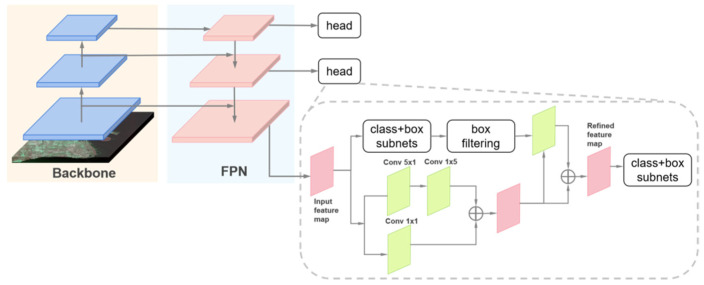
The general architecture of Refined Rotation RetinaNet Detector (R^3^Det). The detection head including a feature refinement module and prediction subnets.

R^3^Det is an end-to-end rotating target detector, using multiscale features output from the backbone to predict proposals. Five parameters (x, y, w, h, θ) predicted by the network represent the center coordinates, width, height, and rotation angle, respectively. To achieve better positioning accuracy and performance, a feature refinement module (FRM) [67] was designed and added after the preliminary prediction of RetinaNet [35], as displayed in Figure 10. The FRM used interpolation to re-encode the current boundary box location information into finer feature points. Then, the feature images were reconstructed and aligned. In the refined stage, only the points with the highest score were retained in each anchor; this measure speeds up the inference meanwhile ensuring each anchor corresponds to only one refined box.

#### 3.1.2. YOLOv4

**Figure 11:**
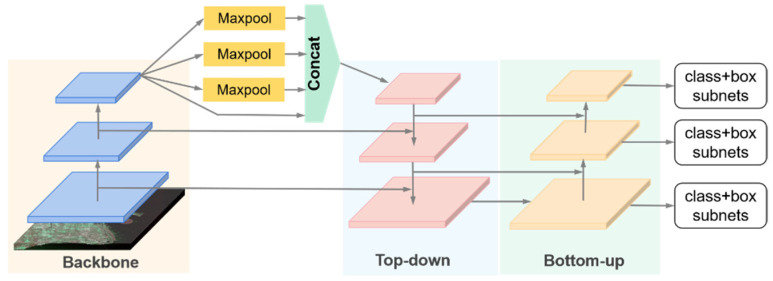
The general architecture of YOLOv4.

The author of YOLOv4 introduced the concepts of “Bag of Freebies” (BoF) and “Bag of Specials” (BoS), which refer to strategies or modules and other training tricks that increase slightly the inference cost but can greatly improve the accuracy of object detection [34]. YOLOv4 adopts those optimization strategies associated with original YOLO architecture in various aspects. In terms of network frameworks which is shown in Figure 11, CSPDarknet53 [34] was chosen as a backbone, the SPP module [33] was added to expand the receptive field, and PANet acted as the measure of aggregation parameters. Other BoF and BoS such as Weighted-Residual-Connections (WRC) [71], Cross-Stage-Partial-connections (CSP) [38], Self-adversarial-training(SAT) [34], Mish Activation [72], Mosaic data Augmentation [34], CIoU Loss [73] were added to YOLOv4.

### 3.2. Implementation Details

All the Detectors were trained on an Ubuntu 16.04 operating system and one NVIDIA GTX 1080Ti GPU with 10 GB memory. As for hyperparameter choice, the momentum and weight decay were set to 0.9 and 0.0001; the batch size was set to 32 and finished in 50 epochs; the Intersection of Union (IoU) threshold was set to 0.7 when training, and valued in the range of 0.5 to 0.95 at 0.05 steps when testing; the NMS threshold was set to 0.3, and the remaining hyperparameters kept the default values during the practical implementation. The training samples were flipped up–down, left–right, and rotated 180° for data augmentation merely in R^3^Det, as YOLOv4 has built-in augmentation tricks. Evaluation metrics including IoU, precision rate, recall rate, and average precision (AP) were employed as indicators to evaluate the performance of the detector quantitatively and comprehensively. IoU was formulated in (4) representing the ratio of the intersection area and union area between the predicted boundary box $Box_{pd}$ and ground truth (GT) boundary box $Box_{gt}$. The higher the IoU was, the better performance the detector achieved.

(4) $$IoU=\frac{Box_{pd}\cap^{\text{​}}Box_{gt}}{Box_{pd}\cup^{\text{​}}Box_{gt}}$$

Precision rate and recall rate were defined as (5) and (6), where TP (True Positives), FP (False Positives), and FN (False Negatives) denote the number of correctly detected targets, wrong targets, and omitted targets, respectively. The precision rate represents how many predictions out of all detected targets were correct; the recall rate represents how many objects out of all real objects were detected. We denote precision rate and recall rate as P and R in the following. Considering the RBox is more difficult than BBox in regression, we took different evaluation criteria as a tradeoff. To be specific, we gave P and R the results of R^3^Det and YOLOv4 with IoU thresholds of 0.3 and 0.5, respectively. By giving different confidence thresholds, we could depict a precision–recall (PR) curve corresponding to a set of confidence thresholds, and compute the area under the PR curve, as known as AP. The larger the AP was, the higher the average accuracy of the detector. Following the evaluation metrics in MS COCO, we calculated the value of AP0.5 and AP0.5:0.95 when testing. The former was the AP on the condition that the IoU threshold was 0.5, and the latter was the mean value of ten AP, whose IoU thresholds ranged from 0.5 to 0.95 with the step of 0.05.

(5) $$Precision=\frac{TP}{TP+FP}$$

(6) $$Recall=\frac{TP}{TP+FN}$$

### 3.3. Experimental Results

In this section, detection results of the two networks on DSSDD are presented. In order to confirm the effectiveness of pseudo-color enhancement, a comparison was made with the images comprised of only a single polarization channel.

VV and VH polarized images were treated as control groups, their contents were in full accord except for pixel values. For R^3^Det, two basic networks ResNet50 and ResNet101 [74] concatenated with FPN were chosen as the backbone. In consideration of the difficulty in RBox regression, only positive samples whose IoU rate with groundtruth over 0.3 were regarded as true objects. Table 2 indicates that the network using the pseudo-color images had better P and R than that with only VV or VH polarized data. We observed that the accuracy of the ResNet50 model trained with different algorithms was slightly lower than that of the ResNet101. We speculate that a possible reason is that a deeper network can better fit object characteristics, which has been shown by studies in other papers. Despite this, the pseudo-color enhancement showed significant promotion properties. Specifically, AP0.5:0.95 for the VV and VH modes was elevated by about 10.1% and 6.1% under the ResNet50 framework, and correspondingly under ResNet101 framework, 3.5% and 2.9%.

As a deep detector for BBox, YOLOv4 has forceful capability in feature extraction. We used the CSPDarknet53 classifier as the backbone which proved in [34] to be an adequate backbone in YOLOv4. We set the IoU threshold to 0.5 when testing. Unsurprisingly, our dataset achieved the numerically optimal results in terms of P and R rate and AP compared to those of the VV or VH data; the specific numbers are shown in Table 3.

**Figure 12:**
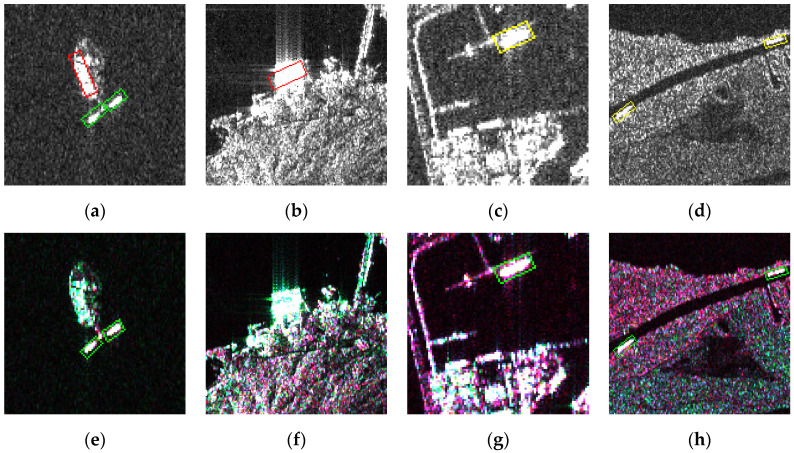
Comparison of RBox detection results on single-polarimetric images (**a**–**d**) and pseudo-color enhanced images (**e**–**h**) of DSSDD.

To further demonstrate the superiority of pseudo-color enhancement in preprocessing the SAR imagery, we plotted some representative results and compared them with single polarization data results in Figure 12. Red boxes represented false positive targets, yellow boxes were for missed targets, and green indicated true positive targets.

Pixels in single-polarized SAR images that corresponded to reflection intensity only differed in brightness, which would cause a noticeable drawback, ambiguity. Coastal land generally has sophisticated environments such as rugged surfaces and metal roofs. Such locations may reflect intense echo in certain radar incident angles and polarization mode, and appear as white speckles on land. Ship-shaped bright spots are confused with real ships, therefore causing false alarms. For instance, the strong reflection areas in Figure 12a,b were mistaken for a ship. The problems mentioned above were mitigated through polarization information fusion. White speckles near the shore depicted in Figure 12a,b had more complete details in Figure 12e,f; hence, the shapes were no longer similar to the real ships. Consequently, the amounts of false alarms were reduced.

There exists another situation where inshore vessels merged into the surrounding backgrounds, which made the outlines of ships indecipherable. Difficulties arise when recognizing the ship shapes whose silhouettes are disturbed by the reflections from the shore. As in Figure 12c,d, ships were ignored for having approximately the same brightness as the land or port. The inability of the network to distinguish between ships and background was one of the main causes of missing detection. Meanwhile, in Figure 12g,h, the background was noticeably darker, and the vague texture of terrain and harbors became clearer. The ships that were previously confused with land in Figure 12c,d were remarkably separated and were detected within green boxes. Different from the detection results on single-polarized images, the identification results of DSSDD were more accurate.

In sum, pseudo-color enhancement can overcome the limitations of the SAR mechanism to a certain extent and improve the detection accuracy. Essentially, pseudo-color images fuse different polarization characteristics. In this way, they can effectively enhance the target shape, widen the gap between ships and land, and restrain inshore false alarms and missing targets accordingly.

## 4. A Weakly Supervised Method

### 4.1. Motivation

Although the current deep learning approaches have achieved great success, one major drawback is that the cost of data annotation is heavy; thus, it is difficult for many tasks to obtain massive and thorough hand-labeled supervision information [75]. Furthermore, extremely deep hierarchy and complex structure constitute powerful networks that require up to millions of hyperparameters. As a result, such networks rely on high-performance GPU equipment and many hours of training, which exacerbate its time cost and computational complexity. In practical projects, CNN-based detectors sometimes might not be the best choice.

To overcome the shortages mentioned, we propose a weakly supervised anomaly detection method. In contrast to end-to-end networks that predict objects directly, we use reconstruction-based anomaly detection to filter false alarms after CFAR. Anomaly detection aims to find anomalies that have different patterns from the training set, i.e., outliers. In this paper, the anomaly refers to the false alarm, it could be land, sea, noise, etc. False alarms have too many kinds to enumerate; simple dichotomies are unsuitable for this situation. By contrast, fitting and recording the characteristics of positive samples are more intuitive. That is why we do not care about the feature extraction of anomalies but positive samples instead. So only normal samples are available as training data [76]. Autoencoder (AE) only fits and records the characteristics of ships. From doing so, the labeling workload would be quite reduced.

### 4.2. Overall Scheme of Proposed Method

**Figure 13:**
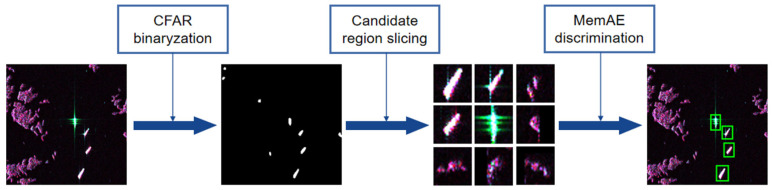
Overall detection procedures of our proposed method.

The whole detection procedure is diagrammed in Figure 13 with three stages included: CFAR binarization, candidate region extraction, and anomaly discrimination. In the first stage, two-parameter CFAR [18] was adopted to binarize the input original image, and then obtain preliminary target region proposals containing false alarms. Each pixel was identified as signal (target) and noise (background) under a constant false alarm rate. In the second stage, white speckles in the binary mask were considered as where the candidate targets were located and were cropped as 28 × 28 size chips for the next stage input. In the last phase, the MemAE [56] was ameliorated and introduced to detect the anomalies. With inference, the autoencoder tends to reconstruct normal results. Theoretically, anomalies produce higher reconstruction errors than normal inputs. In other words, the similarity of the anomaly and its reconstruction result is lower. Therefore, cosine similarity between reconstruction and input was applied as an indicator of false alarm, and an adaptive threshold was selected through the Otsu method [77]. Chips with a similarity less than the threshold were classified as false alarms, while those whose similarity was greater than the threshold were categorized as targets and reserved.

We refer to this method as a weakly supervised approach for the following factors: In the third stage, the chips input to AE were obtained by the traditional image processing method in the first and second stages. When training AE, only false alarm chips needed to be manually removed. When screening out anomalies, the labeling workload was greatly reduced owing to most chips of the CFAR detection results being correctly classified as ships. As for the object detection tasks, we skipped the labeling of location prior information, i.e., the BBox. Thus, we defined this method as weakly supervised learning.

### 4.3. Two-Parameter Constant False Alarm Rate

The constant false alarm detection technology refers to the technology that distinguishes between signal and noise and determines targets based on the statistical characteristics of background clutter while keeping the false alarm probability constant. The core idea was to estimate the power of the background clutter by modeling the sampled data in the background window.

**Figure 14:**
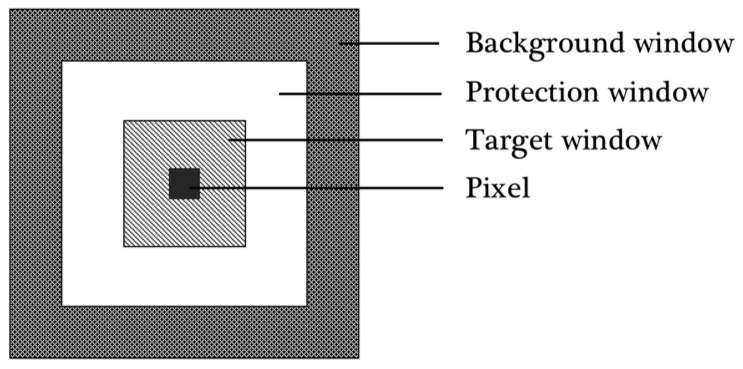
Relationships of three different sliding windows in two-parameter CFAR.

Two-parameter CFAR, which can generally adapt to the change of background clutter, is one of the commonly used classic detection algorithms. Compared with the deep neural network, this method can sensitively detect changes of a small target signal not limited by the receptive field, which is especially useful for our data set. For each pixel in a two-dimensional image, the dual-parameter CFAR established three pixel-centered local sliding windows with customizable sizes, namely, target window T, protection window G, and background window B. Three windows are shown in Figure 14. In the target window were the pixels to be detected; the pixels in the background window were used to calculate Gaussian statistics of sea clutter; the function of the protection window was to ensure that ships would not be included in the background window. In this paper, the sizes of T, G and B were set as 3 × 3, 16 × 16, 32 × 32 respectively.

The criteria for judging ship signals in the two-parameter CFAR is given in (7):

(7) $$Pixel=\{\begin{array}{c} \begin{array}{cc} 1, & \frac{\mu_{T}-\mu_{B}}{\sigma_{B}}\geq thr \end{array}\\ \begin{array}{cc} 0, & \frac{\mu_{T}-\mu_{B}}{\sigma_{B}}<thr \end{array} \end{array}$$

where $\mu_{T}$ denotes the mean value of pixels in the target window, $\mu_{B}$ and $\sigma_{B}$ denote the average and standard deviation of pixels in the background window, and thr is the false alarm threshold, also known as the normalization factor. During the process, sliding windows with a certain step traverse the whole image and return an input-sized binary mask in element-wise comparison.

**Figure 15:**
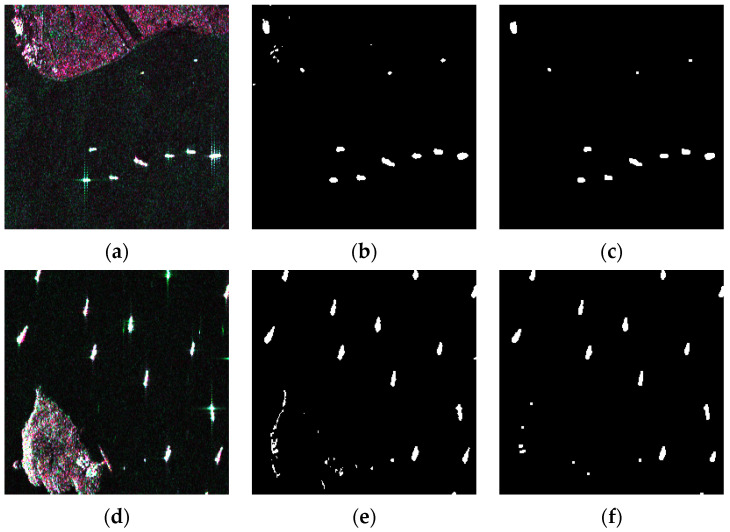
Generation process of binary masks. (**a**,**d**) are two examples of input; (**b**,**e**) are binarization results of two-parameter CFAR and become (**c**,**f**) after morphological transforms.

Figure 15a,d are two examples of input and Figure 15b,e illustrate their binarization results. It can be seen that the results were contaminated by the interference of land. Considering the existence of irregular noise, morphological transforms such as erosion and dilatation were taken after binarization to eliminate the false alarms caused by noise. Figure 15c,f are masks after morphological transforms.

### 4.4. Memory-Augmented Deep Autoencoder

**Figure 16:**
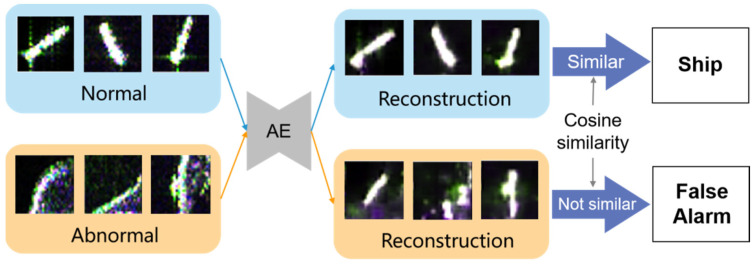
Schematic diagram of an autoencoder for anomaly detection.

Deep autoencoder is a data-specific, lossy, sparse representation method that automatically learns from samples, which has been prevalently used in the anomaly detection field. It consists of an encoder that compresses input data into low-dimensional hidden variables, and a decoder that restores the image from the hidden variable of the hidden layer. Figure 16 explains how the AE discriminates anomalies. AE learns patterns of positive samples and restores input images. It mainly relies on an assumption that anomalies cannot be reconstructed well; the reconstruction error of anomalies is much larger than that of a normal target. Cosine similarity measures the difference in pixels between input and output; hence, it was used to classify input slices into ship category and false alarm category.

However, the AE has too strong an ability of generalization in that sometimes anomalies can be represented after restoration, resulting in the inability to recognize anomalies properly [56]. We applied the MemAE proposed by Gong et al. to alleviate the disadvantage of strong generalization. The key point is that, given an input, MemAE does not feed the encoded variable directly into the decoder but takes it as a query to retrieve the most relevant items in its memory, which are later delivered to the decoder after a combination of weighting.

When screening anomalies, Gong et al. failed to take into account the effect of target size on the reconstruction error. The L2-norm-based mean square error (MSE) formulated in (8) is an absolute error. It adds up every Euclidean distance between pixels of input x and reconstruction $\hat{x}$, which means the reconstruction error is closely related to the number of object pixels with high intensity.

(8) $$MSE\left(x,\hat{x}\right)=||x-\hat{x}{\left|\right|}_{2}^{2}$$

During the experiments, we found that even if a large target was well reconstructed, still a high MSE was produced as each ship pixel introduced some errors. In contrast, some anomalies that consisted of a few high-brightness pixels tended to have a small MSE even if the restoration results were not similar. Considering this situation, we abandoned MSE and adopted cosine similarity as an indicator to discriminate anomalies. The cosine similarity is defined as d(,) in (9). It estimates the relative differences of vectors by measuring the cosine of the angle between them. The output score ranges from 0 to 1. The closer the cosine value is to 1, the more similar the two vectors are.

(9) $$d\left(x,\hat{x}\right)=\frac{x\cdot {\hat{x}}^{T}}{\left||x||\cdot ||{\hat{x}}^{T}|\right|}$$

**Figure 17:**
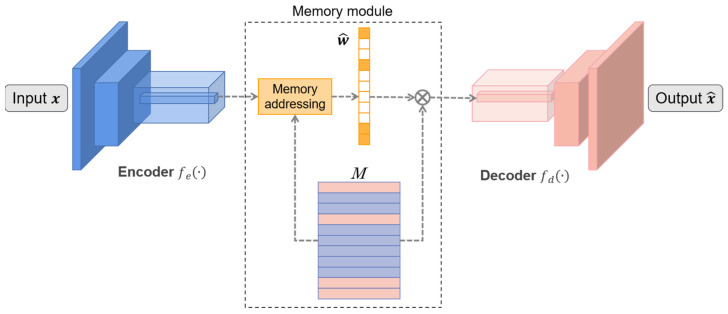
Diagram of memory-augmented deep autoencoder (MemAE).

Figure 17 is the schematic diagram of the restoration procedure. The encoder denoted as $f_{e}\left(\cdot \right)$ is enacted by three convolutional layers, and the decoder denoted as $f_{d}\left(\cdot \right)$ is corresponding to three deconvolution layers. Given a certain size input x, the encoder maps it to encoding z. The memory module is a matrix denoted as M to specifically store the representative normal patterns. It contains N storage units, and the *i*-th storage unit is denoted as $m_{i}$. An addressing scheme was introduced after the encoder so as to find related stored items in M. In detail, a non-negative soft addressing weights w was constructed in (10), where $w_{i}$ denotes the *i*-th entry of w:

(10) $$w_{i}=\frac{\exp\left(d\left(z,m_{i}\right)\right)}{\sum_{j=0}^{N}\exp\left(d\left(z,m_{j}\right)\right)}$$

To ensure the sparsity, w is activated, which means $w_{i}$ only worked if greater than the threshold λ, otherwise 0:

(11) $${\hat{w}}_{i}=\{\begin{array}{cc} w_{i}, & if\;w_{i}>\lambda \\ 0, & otherwise \end{array}$$

In addition, the latent representation $\hat{z}$ is derived according to (12) and delivered into the decoder to reconstruct the $\hat{x}$.

(12) $$\hat{z}=\hat{w}M=\sum_{i=1}^{N}{\hat{w}}_{i}m_{i}$$

### 4.5. Implementation Details

#### 4.5.1. Slicing

Before we conducted training, we first prepared the training set sliced from our pseudo-color images. After the CFAR processing, we calculated the contours and center coordinates of white speckles on binary maps and then executed the slice operation. According to the statistics in Section 2.5, most BBoxes of ships contained less than 800 pixels. For the sake of convenience, we cut 28 × 28 size chips centered on targets. As for the targets whose pixels exceeded this size range, we cropped this target along its minimum bounding rectangle and then resized it to 28 × 28.

#### 4.5.2. Training

The encoder and decoder are simple convolutional neural networks with their parameters shown in Table 4. The MemAE input was fixed 28 × 28 RGB chips, and the memory size N was set to 100. Conv_i represented the *i*-th convolution layer, Dconv_i represented the *i*-th deconvolution layer. Except for the last Dconv, each layer was followed by batch normalization [78] and a ReLU activation layer.

#### 4.5.3. Threshold Selecting

The reconstruction similarity of normal targets was concentrated around 0.9, significantly larger than that of abnormal reconstruction similarity, which was about 0.7. There was an obvious valley point between their intersection. As a classical segmentation technique, the Otsu method could select the threshold near the valley point. This method obtains a global adaptive threshold $t_{otsu}$ by maximizing the between-class variance:

(13) $$t_{otsu}=\underset{0<t<1}{\mathrm{ArgMax}}\left\{\sigma_{B}^{2}\left(t\right)\right\}$$

(14) $$\sigma_{B}^{2}\left(t\right)=\omega_{1}\left(t\right)\omega_{2}\left(t\right){\left(\mu_{1}\left(t\right)-\mu_{2}\left(t\right)\right)}^{2}$$

Class 1 and class 2 denote negative and positive, respectively, in terms of Formula 14. Samples whose cosine similarity was less than the threshold t were categorized as negatives, and those samples with cosine similarity greater than t were categorized as positives. $\sigma_{B}^{2}$ was the between-class variance of the two categories at threshold t, $\omega_{i}\left(t\right)$ was the occurrence probability of *i*-th category at threshold t, and $\mu_{i}\left(t\right)$ was the average cosine similarity of the *i*-th category at threshold t. By traversing all t, we can find the threshold $t_{otsu}$ when the variance between classes was maximum. Chips whose cosine similarities were less than $t_{otsu}$ were considered as false alarms and thereby abandoned.

### 4.6. Results Analysis

**Figure 18:**
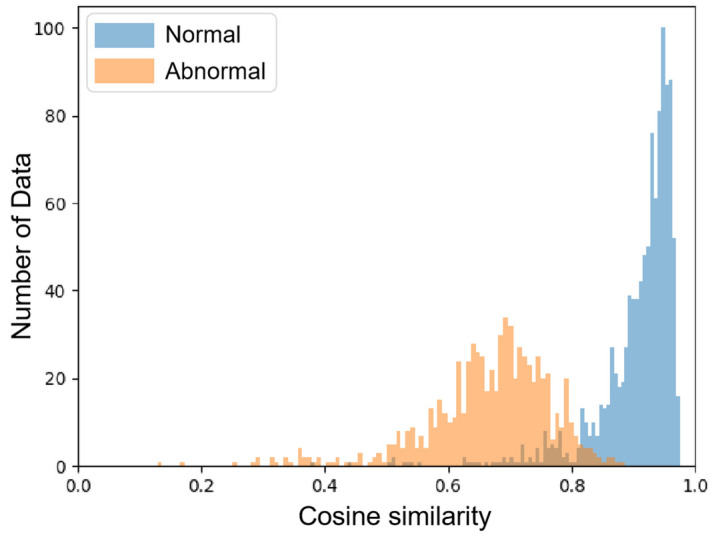
The distribution of cosine similarity between input and restored chips. Ship chips and false alarms are indicated in blue and orange respectively.

The experiments were conducted on our DSSDD. We used three evaluation indexes P, R, and AP to verify the effectiveness of our method. The cosine similarities of the testing set are visualized in a histogram, Figure 18; ships and false alarms are represented by blue and orange bins respectively. The $t_{otsu}$ was around 0.8 in the test set, with which most of the testing chips could be classified properly.

**Figure 19:**
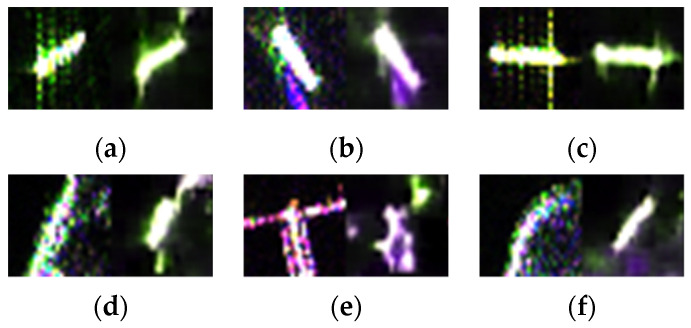
Visualization of reconstruction results. (**a**–**c**) are comparison of real ship chips on the left and images restored from MemAE on the right. On the left of (**d**–**f**) are false alarms and on the right are the corresponding reconstructions.

Some representative chips restored from MemAE were visualized as below and compared with the original inputs. On the left side of Figure 19a–c are real ship objects, and reconstructed images on the right side. Figure 19d–f are inputs and outputs of false alarms. The visualization results further confirmed positive samples could be restored well, while anomalies’ reconstruction errors were large, which was consistent with our expectation.

**Figure 20:**
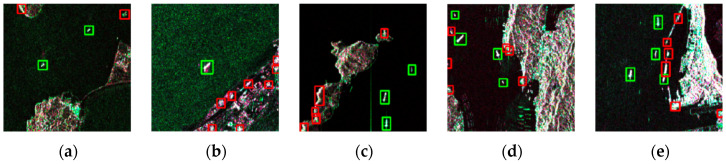

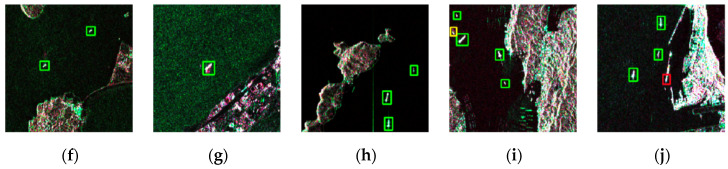
Illustration of detection results where red boxes represent false alarms, yellow boxes are missed ships, and green boxes are correct ships. (**a**–**e**) are detection results of two-parameter CFAR; (**f**–**j**) are detection results of our method that adds the MemAE module. Our method significantly screened false alarms by using an anomaly detection approach with acceptable recall loss.

Table 5 displays the testing results of using only two-parameter CFAR as well as that of our method. The comparison of these two results is revealed in Figure 20, where red boxes represent false alarms, yellow boxes are for missed ships, and green ones indicate true positive targets. It can be seen from the table that CFAR detection would introduce a large number of false alarms while achieving a high recall rate; the precision was only 0.773. Beneficial from the filtering of MemAE, the nearshore false alarms were suppressed to a large extent, and the precision was increased by 15.3% meanwhile, reaching a 0.923 recall rate. It is commonly a tradeoff between precision and recall.

To further argue for the performance of proposed method, three additional pytorch version of CNN detectors, i.e., EfficientDet-D0, YOLOv4-tiny, and MobileNetV3 with SSD heads were conducted on DSSDD. We evaluated comparative detectors from six aspects: AP under 0.5 IoU threshold; *P*, *R* under F1 point; Parameters; FLOPs; and speed of once inference on Intel Xeon CPU. Comparative experiments are displayed in Table 6, where *M* and *B* refer to million and billion respectively.

The results demonstrate that our proposed detection method can achieve an equivalent effect to the deep neural network, while the parameters and FLOPs are the lowest of all. We found that MobileNetV3 had the fastest speed but had poor performance on our dataset as well.

In general, the proposed method combines the advantages of conventional algorithms and CNN-based detectors. This method occupies little memory and is easy to train, which makes it a lightweight model. It does not require large-scale datasets or high-performance hardware devices and can be transplanted to different devices and generalized to various practical engineering projects. We hope that the proposed method can provide some inspiration and help to other scholars and fields of research.

## 5. Conclusions

In this study, a dual-polarimetric SAR ship detection dataset DSSDD containing 1236 ship slices was constructed. The baselines of DSSDD were established on two SOTA models. The experimental results show our pseudo-color enhanced images are superior to single-polarized data in emphasizing objects, avoiding ambiguity, and fusing characteristic information. Our preprocessing method can effectively improve detection precision and recall rate. In addition, a weakly supervised method combining two-parameter CFAR with autoencoder was proposed. To eliminate false alarms generated by the CFAR method, we introduced an advanced memory-augmented deep autoencoder. By calculating the reconstruction similarity, we can effectively identify false alarms detected from CFAR. Experiments were carried out on DSSDD. With acceptable cost, this method has shown performance comparable to supervised learning, making it a promising direction for weakly supervised ship detection. In future work, we will focus on further improvement of the model structure as well as the detecting performance, aiming at promoting the development in the SAR ship detection field. 

## Figures and Tables

**Table 1:** Detailed information for the original Sentinel-1 imagery.

Satellite	Imaging Mode	Resolution Rg. × Az.(m)	Swath (km)	Polarization Modes	Incident Angle (°)	Product Type	Number of Images
Sentinel-1	IW	2.3 × 14.0	250	VV + VH	29.1~46.0	SLC	50

**Table 2:** R^3^Det detection results on pseudo-color enhanced and corresponding VV and VH polarized test set of DSSDD.

Method/Dataset		Backbone	Precision	Recall	AP0.5	AP0.5:0.95
R^3^Det	VV	ResNet-50 + FPN	0.936	0.893	0.888	0.304
	VH		0.942	0.877	0.887	0.334
	Pseudo-color		0.957	0.921	0.902	0.405
	VV	ResNet-101 + FPN	0.943	0.913	0.899	0.440
	VH		0.946	0.903	0.896	0.446
	Pseudo-color		0.962	0.915	0.902	0.475

**Table 3:** YOLOv4 detection results on pseudo-color enhanced and the corresponding VV and VH polarized test set of DSSDD.

Method/Dataset		Backbone	Precision	Recall	AP0.5	AP0.5:0.95
YOLOv4	VV	CSPDarknet53	0.944	0.923	0.924	0.579
	VH		0.948	0.922	0.922	0.551
	Pseudo-color		0.958	0.933	0.938	0.585

**Table 4:** Convolutional structure and parameters of the encoder and decoder in MemAE.

Layer Name	Output Size	Kernel Size	Stride
Input	28 × 28	-	-
Conv_1	14 × 14	3 × 3, 16	2
Conv_2	7 × 7	3 × 3, 32	2
Conv_3	4 × 4	3 × 3, 64	2
Dconv_1	7 × 7	3 × 3, 32	2
Dconv_2	14 × 14	3 × 3, 16	2
Dconv_3	28 × 28	3 × 3, 3	2

**Table 5:** Detection precision and recall rate of both two-parameter CFAR and our method. Compared with the former approach, ours removed more false alarms.

Method	Precision	Recall
CFAR	0.773	0.966
Ours	0.926	0.923

**Table 6:** Results of different detectors on DSSDD. Our method has a lightweight detector and can achieve comparable performance with CNN-based detectors.

Method	*P*	*R*	AP	Params	FLOPs	Speed
EfficientDet-D0	0.918	0.887	0.911	3.9 M	2.5 B	366 ms
YOLOv4-tiny	0.933	0.924	0.926	5.9 M	3.4 B	176 ms
MobileNetV3 + SSD	0.874	0.843	0.837	2.7 M	420 M	64 ms
Ours	0.926	0.923	0.925	1.5 M	1.4 M	82 ms

## Data Availability

Not applicable.
